# Melt Conditioning of Light Metals by Application of High Shear for Improved Microstructure and Defect Control

**DOI:** 10.1007/s11837-017-2335-5

**Published:** 2017-04-07

**Authors:** Jayesh B. Patel, Xinliang Yang, Chamini L. Mendis, Zhongyun Fan

**Affiliations:** The EPSRC Centre – LiME Hub, BCAST, Brunel University London, Uxbridge, UB8 3PH UK

## Abstract

Casting is the first step toward the production of majority of metal products whether the final processing step is casting or other thermomechanical processes such as extrusion or forging. The high shear melt conditioning provides an easily adopted pathway to producing castings with a more uniform fine-grained microstructure along with a more uniform distribution of the chemical composition leading to fewer defects as a result of reduced shrinkage porosities and the presence of large oxide films through the microstructure. The effectiveness of high shear melt conditioning in improving the microstructure of processes used in industry illustrates the versatility of the high shear melt conditioning technology. The application of high shear process to direct chill and twin roll casting process is demonstrated with examples from magnesium melts.

## Introduction

Light alloys based on aluminum and magnesium are used in many industrial applications where light weighting is of paramount interest.^1^ This is especially true in the automotive sector where light weighting transfers into reduced CO_2_ emissions and increased fuel efficiencies. The light weighting has become more important for increased fuel efficiencies, with the increase in the weight of automobiles as a result of the weight of batteries or fuel cells associated with electric vehicles. The development of high-strength alloys and composites requires a refined microstructure with a reduced defect distribution together with a homogeneous composition profile in the final product. Solidification and casting is essential for processing metallic materials regardless of whether the final product is used in the cast or wrought form. The quality of the casting and in turn the quality of the melt is crucial in determining the final properties. Oxides, gas and other inclusion usually deteriorate the quality of the melt and in turn the properties and quality of the castings. The presence of micro- and macro-scale defects, such as gas porosity, and oxide defects in the solidified billets can lead to reduced property profiles in castings and wrought products. The presence of pores and other defects would reduce the strength and ductility of the castings. Thus, the yield strength used for design process is significantly lower than the average yield strength of the alloy to account for these defects. In applications where light weighting is of importance, such increments reduce the weight savings envisaged and, in the case of automotive or aerospace applications, reduce fuel efficiencies and consequently increase the CO_2_ emission. The reduction in defect density and the resultant increase in strength would contribute to the lighter parts, and this may be achieved through melt conditioning, especially with intensive melt shearing.

Melt treatment ensures a high-quality melt through treating the liquid metal prior to casting. There are numerous existing methods including electromagnetic stirring, mechanical stirring with an impeller, melt filtering, and rotary degassing, which are used to treat the liquid metals. Recently, melt conditioning of Al and Mg melts in liquid and semisolid states, using a twin-screw device, refined the microstructure and improved the mechanical properties of both cast and wrought alloys.^2^–^7^ Due to the size of the twin screw device, the incorporation of this method into the existing processing chain is difficult. Thus, a simpler way to incorporate the melt shearing technique into a process is required, leading to the development of a new technology based on a rotor–stator shearing mechanism.^8^ The rotor–stator device developed within Brunel Centre for Advanced Solidification Technology provides intensive melt shearing, dispersing inclusions into finer scale particles that enhance the number of nucleation sites. The experimental results to date show that this high shear device could be used for general melt treatment, grain refinement, degassing, incorporation of particles into metal-matrix composites, and refining semisolid slurries. In this contribution, we present an overview of the application of the high shear technology using the new rotor–stator device in light metals.

### Principles Associated with Rotor–Stator Shearing Mechanism

**Fig. 1:**
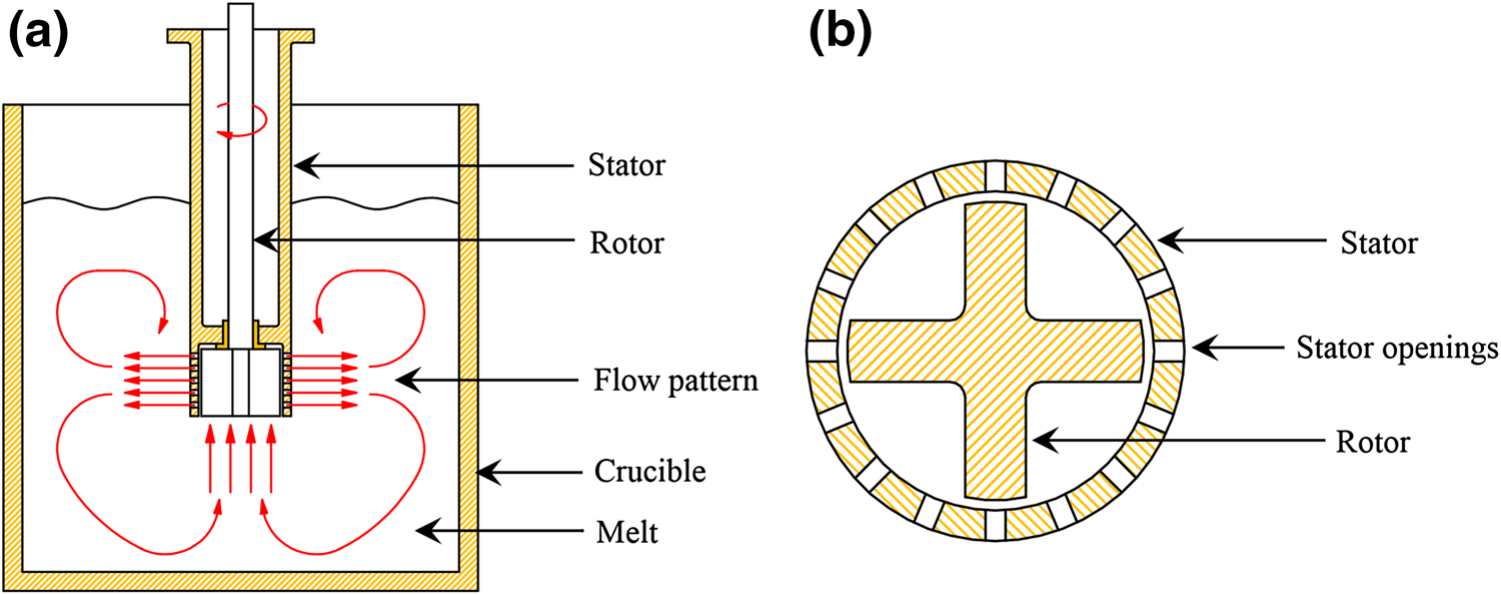
Schematic illustration of the rotor stator high shear mixer (a) transverse cross section illustrating the melt convections and (b) bottom view of the high shear mixer

A schematic representation of the rotor–stator device is shown in Fig. 1. The device consists of a rotor and a stator with a speed control attached to an electrical motor. During operation, rotation of the rotor shears the liquid metal in the gap between the rotor and the stator and, additionally, in the openings in the stator. The rotation speed may be adjusted between 5000 rpm and 15,000 rpm to provide a shear rate of up to 10^5^ s^−1^. The rotor–stator high shear device provides macro-flow in a volume of melt for distributive mixing and intensive shearing near the tip of the device for dispersive mixing. The enhanced kinetics associated with chemical reactions and phase transformations, uniform dispersion and reduction of particle size and gas bubbles, uniform distribution of composition and temperature fields, and forced wetting of solid particles in the liquid metal are some of the main advantages associated with the rotor stator. Thus, the high shear device is used effectively for the grain refinement by dispersing native oxides or grain refiners, for degassing aluminum melts, for the preparation of metal-matrix composites, and for preparation the of semisolid slurries. As a result of its size and versatility, the rotor–stator high shear device can be used for many different industrial casting processes for both aluminum and magnesium alloys, including direct chill casting and twin roll casting. The rotor–stator device can be inserted into a crucible containing molten metal and could be used to apply intensive shearing without disturbing the melt surface (Fig. 1). The particles dispersed in the melt can then be intensively sheared to allow uniform dispersion of particulate oxide, grain refiners, and reinforcing particles such as SiC particles throughout the melt.

### Grain Refinement in Light Metals

**Fig. 2:**
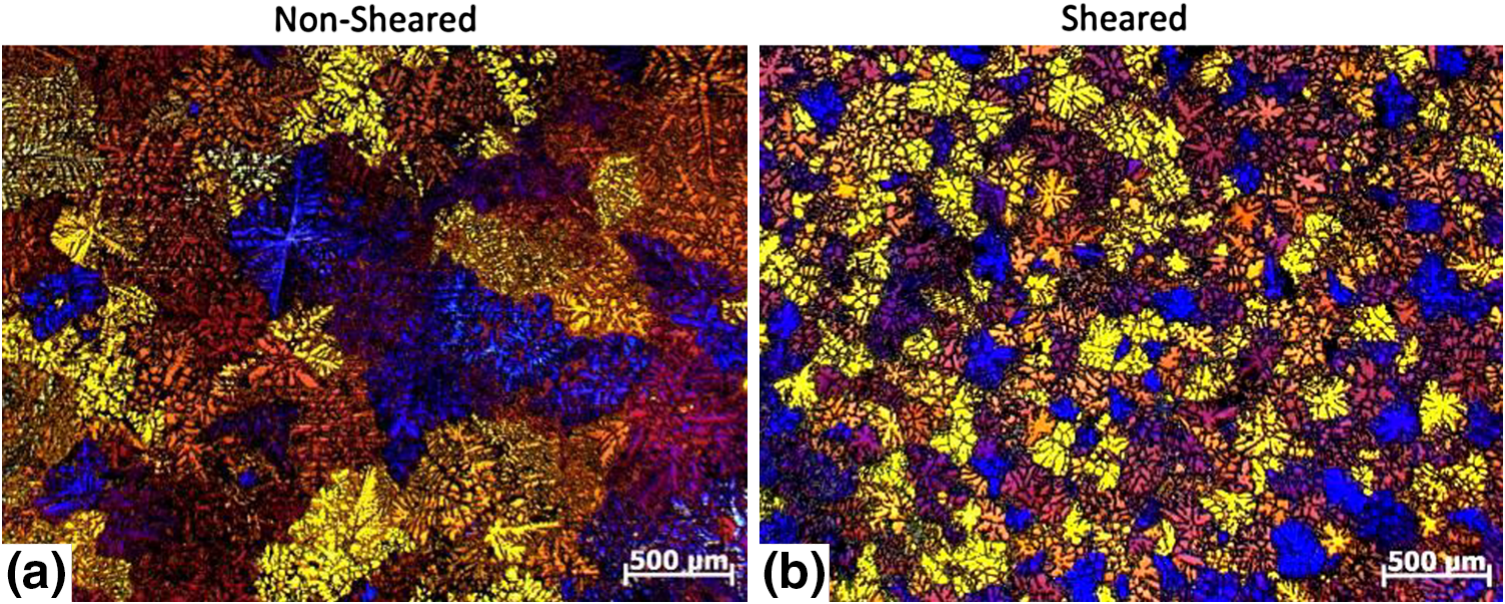
Microstructure of AZ91D magnesium alloy cast at 650°C (a) without melt shearing and (b) with melt shearing

**Fig. 3:**
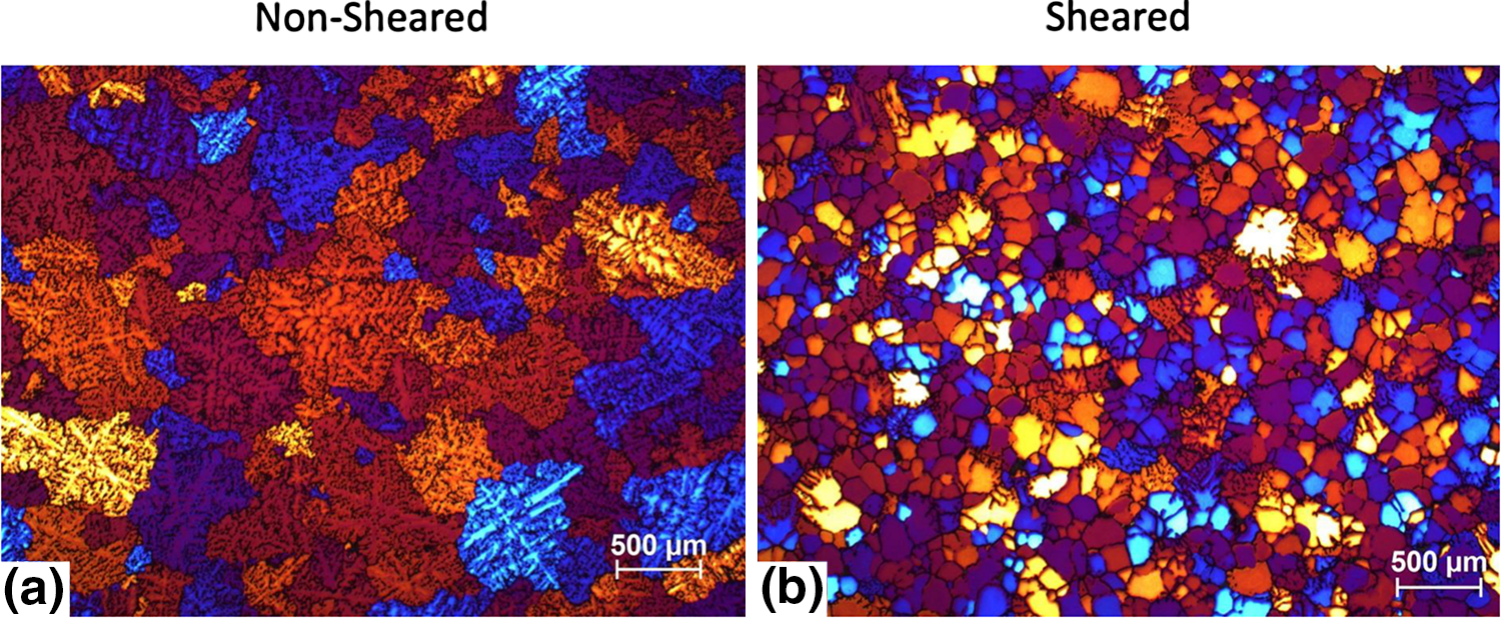
Microstructure of A6082 aluminum alloy cast at 700°C (a) without melt shearing and (b) with melt shearing

The reduction in the defect size and distribution results in enhanced casting soundness and grain refinement. A refined grain size also means a smaller solidification front resulting in reduced solute segregation. The grain refining capability of the high shear technology was demonstrated for magnesium and aluminum alloys using commercial AZ91D and A6082 alloys, respectively, using a standard TP1 test casting.^9^ Figure 2 illustrates the grain refinement caused by high shearing in the AZ91D alloy where the alloy with and without shearing was processed at 650°C where the grain size reduced from approximately 550 *µ*m in the nonsheared alloy (Fig. 2a) to 170 *µ*m in the melt sheared alloy (Fig. 2b). Similar reduction in grain size was observed at lower casting temperatures for the AZ91 alloy illustrating that super heating that is important during casting Mg alloys was reduced significantly, as illustrated by the lower casting temperature of 605°C where the grain size of 180 *µ*m was observed for the nonsheared alloy while the shearing resulted in a grain size to approximately 90 *µ*m. The grain refinement observed in the Mg alloys is attributed to the effective dispersion of MgO films found in the Mg alloys more effective at providing sites for nucleation of Mg grains. The mechanisms associated with the grain refinement of Mg alloys is discussed in detail in Fan et al.^2^ Similar grain refinement to that observed in the Mg alloys was also observed in aluminum alloys with intensive melt shearing as observed for the A6082 aluminum alloy following melt shearing and casting (Fig. 3). In the case of the A6082 alloy, melt shearing changes the typical dendritic microstructure in the nonsheared casting to an equiaxed grain structure observed in the alloy subjected to intensive melt shearing.

### Application of Melt Shearing to the Direct Chill Casting Process

**Fig. 4:**
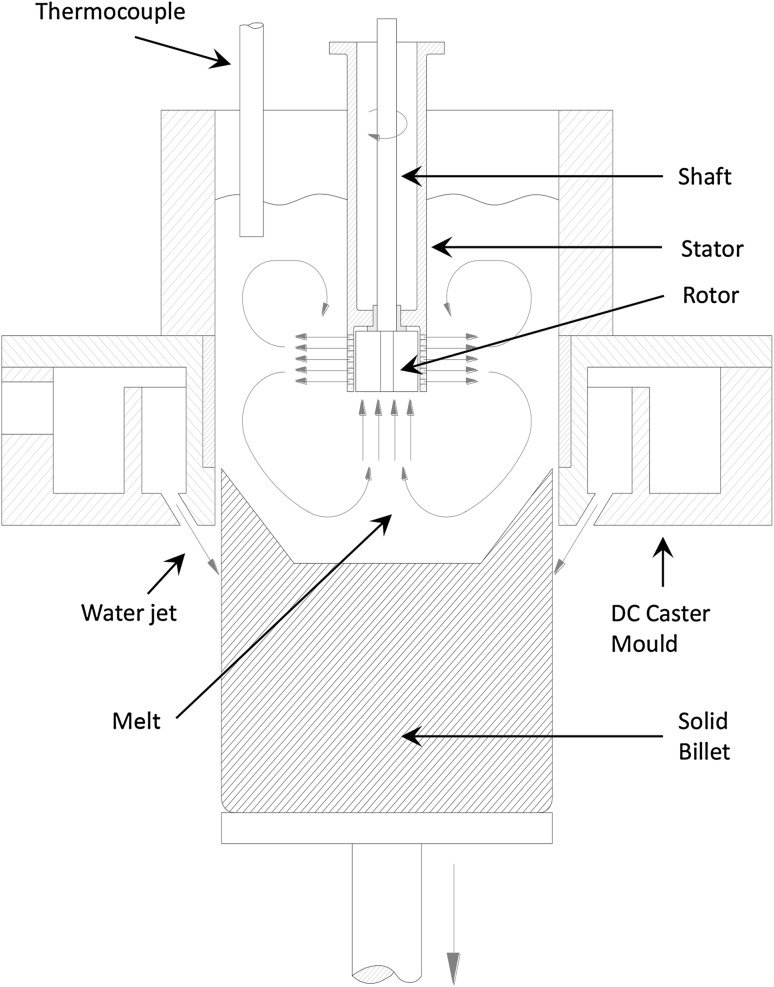
Schematic illustration of the melt conditioned direct chill casting process with the high shear device submerged in the sump of conventional direct chill casting mold. The macroscopic flow paths are also illustrated

**Fig. 5:**
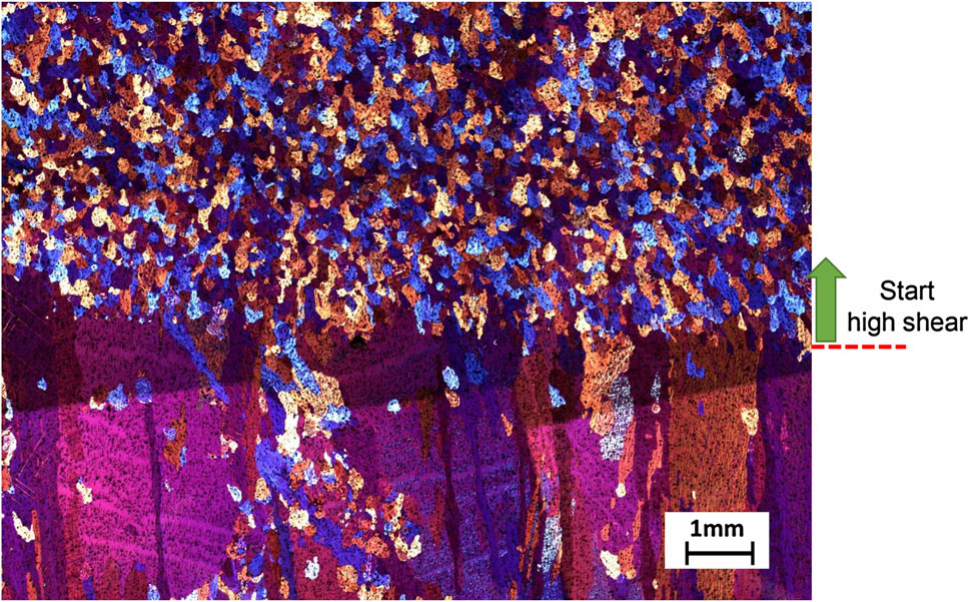
Microstructure of the melt conditioned direct chill (MC-DC) cast AZ31 magnesium alloy billet showing the instantaneous microstructural change from columnar to equiaxed transition upon application of high shear

The melt conditioning on the direct chill (DC) casting process is shown in Fig. 4 where the high shear device is submerged in the sump of a conventional DC mold to provide melt shearing during casting. The high shear device was immersed in the sump of the DC caster, and during the first half of the billet production, high shear was not applied and then the device was turned on during the second half of the billet production to provide high shear. Figure 5 illustrates the changes in microstructural evolution of the DC cast AZ31D alloy cast without high shear (bottom) to one with the high shear (top). Without intensive melt shearing, the DC cast billet contains a coarse columnar microstructure; with intensive melt shearing, the microstructure instantaneously becomes fine and equiaxed. This illustrates that the intensive melt shearing produces a uniform equiaxed grain structure throughout the billet providing a microstructure that is more suited for further processing via extrusion or forging processes. The melt conditioned billet in turn shows more uniform chemical composition from the surface toward the center of the billet compared with conventional DC cast billets. This is attributed mainly to the refined and uniform microstructure throughout the billet.

To understand the grain refining via intensive melt shearing, temperatures were recorded during DC casting by attaching thermocouples to the head of the high shear device.^10^ The direct measurement of the sump depth, during the casting process, showed that the bottom of the high shear device about 40 mm above the interface between liquid and solidified metal. Without melt shearing, the temperature in the sump is nonuniform. When the distance from the solidification front is increased, the observed melt temperature also increased remarkably. Nevertheless, with the high shear, a sharp change in melt temperature distribution in the sump is observed. Intensive melt shearing within results in a more uniform melt temperature throughout the contained volume of melt. The temperature curves showed that during intensive melt shearing, the temperature of the entire volume of melt stabilize very close to the liquidus temperature, eventually reaching a plateau that is slightly lower than the liquidus temperature, especially with increased rotation speed. This provides an important contribution to the grain refinement and reduction or elimination of chemical segregation in the melt conditioned DC casting process.

### Application of Melt Shearing to the Twin Roll Casting (TRC) Process

**Fig. 6:**
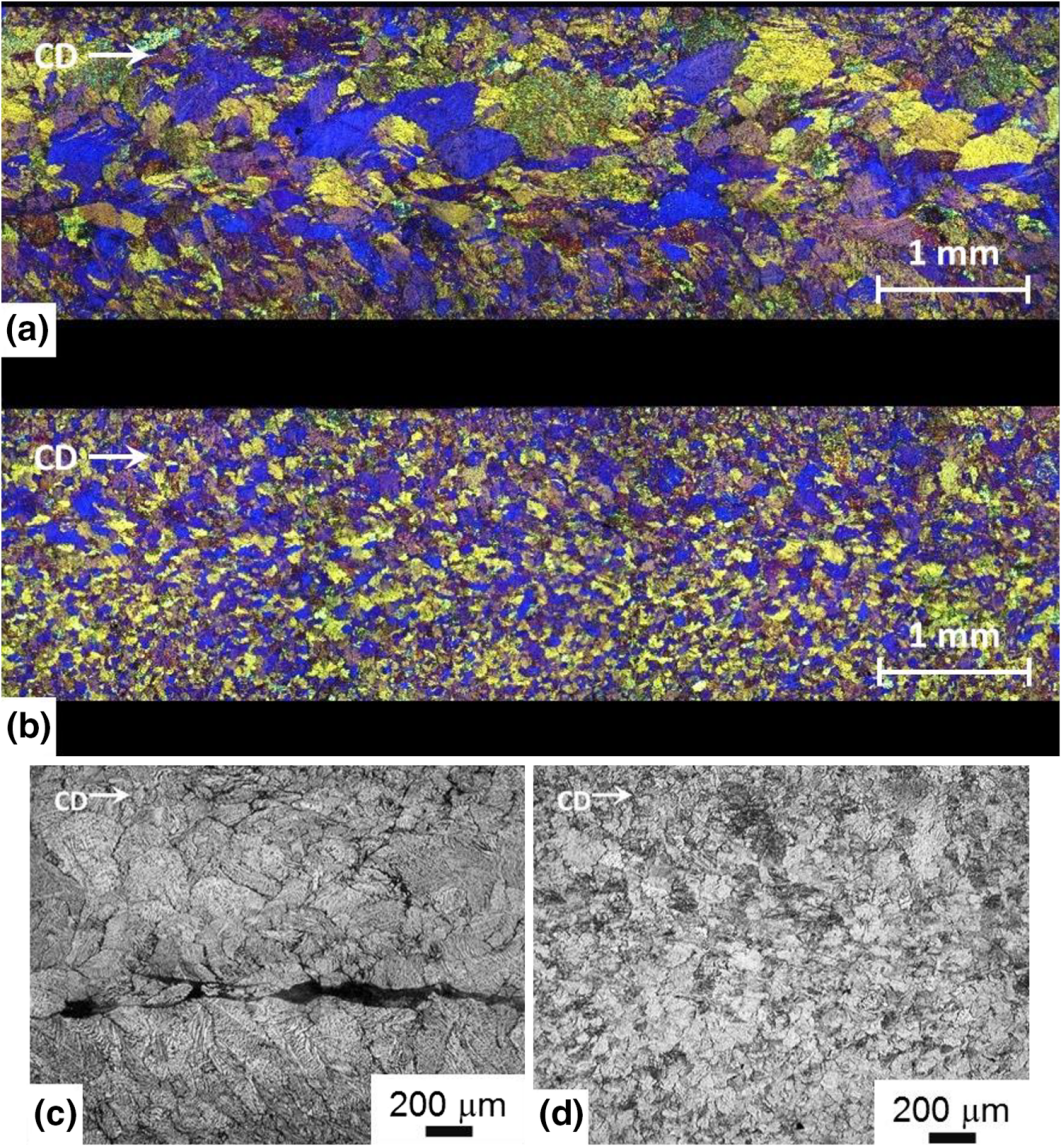
Microstructures of the twin roll cast AZ31 magnesium alloy (a, c) without melt shearing (b, d) and with melt shearing. (a, b) show the grain size distribution across the thickness of the TRC sheet and (c, d) show the center line segregation

Twin roll casting is an important industrial process used in producing magnesium alloy sheets economically. TRC alloys generally contain relatively large grains and contain defects such as center line segregation and inverse segregation along the cast strip. Center line segregation combined with large grain sizes prevent further processing of the TRC sheet without further rolling, which introduce strong basal textures and associated asymmetries between tensile and compressive yield strength. Figure 6 show the microstructure of the TRC (Fig. 6a) and melt conditioned TRC (Fig. 6b) for the AZ31 alloy. The TRC sheet contains large columnar grains with grains meeting at the center line with a fine structure at the surface. Following melt conditioning, the microstructure of the MC-TRC cast strip shows a fine equiaxed grain size with a uniform size distribution through the thickness of the sheet. Intensive melt shearing causes a change in the solidification front during casting resulting in uniform distribution of grains throughout the thickness of the sheet. Figure 6c shows the center line segregation associated with the TRC sheets without intensive melt shearing as the solute is pushed out into the center of the TRC sheet in this region is the last to solidify. The intensive melt shearing results in significant reduction in center line segregation as a result of the smaller solidification front caused by the finer microstructure (Fig. 6d). The uniform distribution of fine grain size results in a uniform composition across the thickness of the melt conditioned TRC sheet producing a finer intermetallic distribution. The finer grain size also allows for easier formability.

### Application of Melt Shearing for Incorporation of Sic Particles into Mg Alloys

**Fig. 7:**
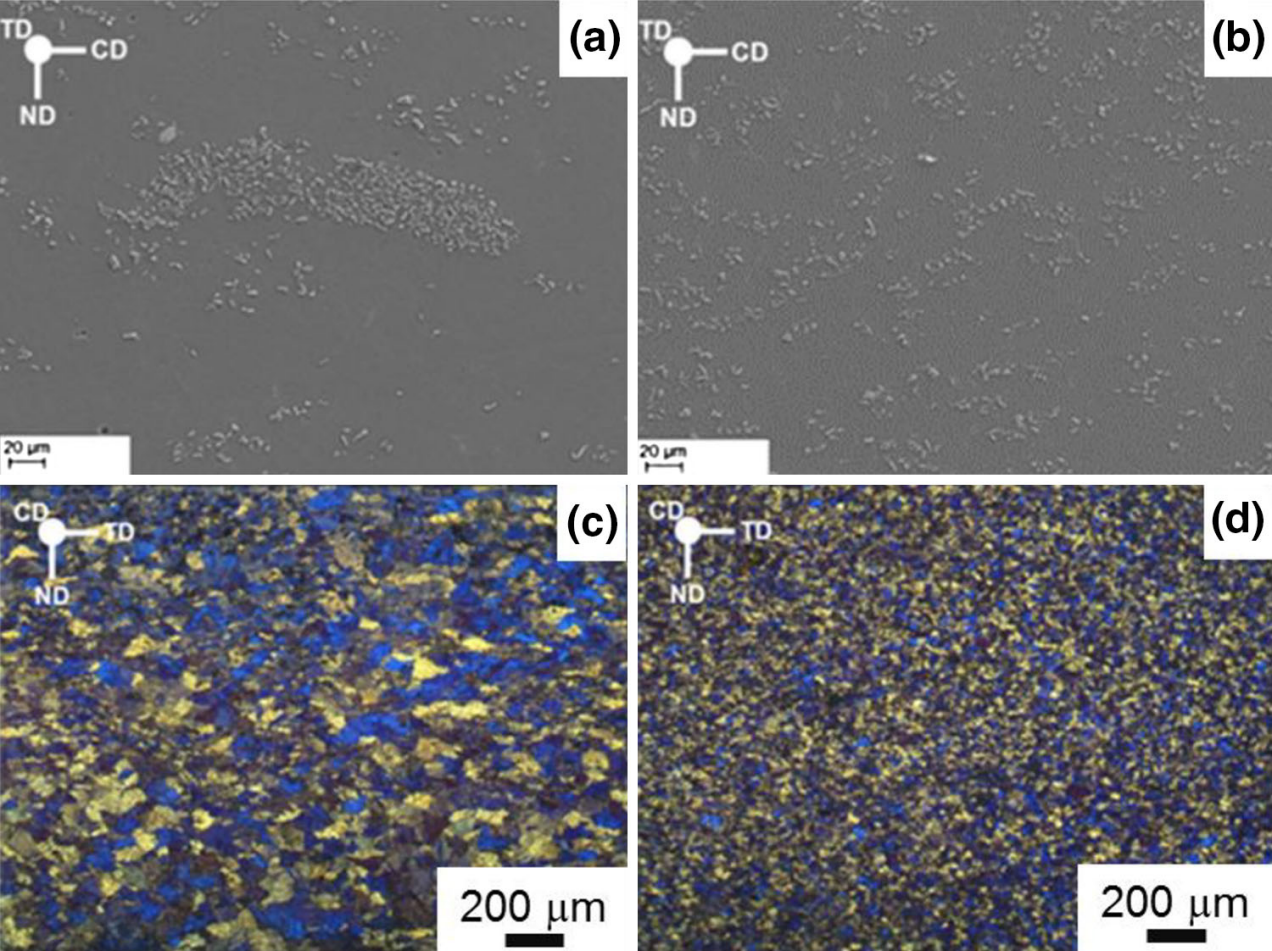
Microstructures of the metal-matrix composites of AZ31 with 5 wt.% SiC (a, c) without melt shearing and (b, d) with melt shearing. (a, b) show the distribution of the SiC particles and (c, d) illustrate the grain size distribution. (Adapted from Ref. 14)

The addition of reinforcing particles to develop high-strength metal-matrix composites has been investigated for the light metals for specialist applications. Achieving a uniform distribution of reinforcing particles in the melt is difficult and mechanical stirring^11^ is used routinely to mix the reinforcing particles in the molten alloys. The problem with conventional mechanical stirring is that the shear rate applied is inadequate to break up particle agglomerations,^12^ particularly when there is a high-volume fraction of particles.^13^ The intensive shearing is suitable for particle dispersion of reinforcement and inoculant in light metals as the high turbulence and high shear rate enhance the breakup of solid particle clusters in liquid medium. AZ31 alloy mixed with 5 wt.% SiC particles was twin roll cast to illustrate the efficiency of intensive high shear in dispersing the reinforcing SiC particles throughout the melt. The microstructures of Mg alloy composite, AZ31 alloy containing 5 wt.% SiC, show a more uniform distribution of the SiC particles when intensive high shearing is applied following impeller mixing as compared with composite only mixed with impeller mixing (Fig. 7a and b). The uniform distribution of the SiC particles also results in the refinement of grain size of the intensively sheared composite, which is significantly smaller than that of the nonsheared composite (Fig. 7c and d). The melt shearing allows for the breakup of agglomerations of particles and allows for the better distribution of the particles that in addition to distributing in the interdendritic regions of the melt provide more sites for nucleation of the magnesium grains. The high shear process is shown to improve the yield, ultimate tensile strength, and the elongation to failure of the AZ31 composites compared with the nonsheared composite.^14^^,^ 15

## Summary

The intensive melt shearing was used to engineer the melt pool through the dispersion of oxides and other inclusions through the melt using a rotor–stator device to achieve the high quality of melt that in turn resulted in improved quality of castings. The rotor–stator high shear device provides distributive and dispersive mixing that significantly enhances the kinetics of phase transformations; improves uniform dispersion, distribution, and size reduction of particles; improves uniformity of composition and temperature in the melt pool; and most importantly, provides physical grain refinement through the dispersion of naturally occurring oxides. Therefore, the rotor–stator high shear device may be used to condition light alloy melts, and it may be implemented with various conventional casting processes such as direct chill casting and twin roll casting.
